# Analyzing the Tensile Creep Behavior of Different Types of Polypropylenes Using a Simple Fractional Differential Viscoelastic Model

**DOI:** 10.3390/polym17081095

**Published:** 2025-04-18

**Authors:** Yasuhiko Otsuki, Kou Hashimoto, Yutaka Kobayashi, Shotaro Nishitsuji, Hisao Matsuno, Hiroshi Ito

**Affiliations:** 1 Research Center for GREEN Materials and Advanced Processing, Yamagata University, Yonezawa 992-8510, Yamagata, Japan; 2 Graduate School of Organic Materials Science, Yamagata University, Yonezawa 992-8510, Yamagata, Japan

**Keywords:** creep, polypropylene, modeling, recycling, fractional differential, viscoelastic model, Eyring rule, Monkman–Grant law

## Abstract

Fractional differential viscoelastic calculus was used to develop a model for predicting the primary to tertiary creep in the tensile creep deformation of various polypropylenes (PPs). The primary and secondary creep were described via simple fractional differential viscoelasticity with an empirical formula for the stress and temperature dependence of the fractional differential order. Tertiary creep was treated as a pure viscous body with damage. The temperature dependence is treated simply, and Arrhenius’s law is applied. As for stress dependence, the Eyring law of the sinh function was applied to the primary and secondary creep processes, while the WLF-type shift function was adopted for tertiary creep. The primary and secondary creep behaviors of each model material showed creep growth rates according to the rigidity of each material. As for the tertiary creep, the homo PP showed a little damage progression with a damage index of 0.17, while the impact-resistant PP showed faster damage progression with a damage index of around 0.5. The three types of post-consumer recycled PPs showed intermediate properties between these virgin PPs, and no peculiarities were confirmed in the static creep behaviors. It was confirmed that the creep experimental results for all model materials fell on the same Monkman–Grant law. The presented creep model can predict the creep strain transition and minimum strain rate well and is effective in predicting the creep characteristics of PPs.

## 1. Introduction

Understanding the creep characteristics of polymeric materials is crucial to ensure the safety and reliability of product structure. In recent years especially, there has been a push to reduce product weight by increasing the stiffness-to-weight ratio as much as possible, due to the need to curb the use of finite fossil resources and reduce CO_2_ emissions, both closely associated with global warming. This demand has led to a trend in many fields toward reducing the thickness of products, and, as the load stress on products has tended to increase, it has become necessary to carefully design products with resistance to creep deformation. Furthermore, in recent years, the problem of waste pollution has become more serious, and material recycling has been promoted worldwide to create a sustainable recycling-oriented society [1,2]. Material recycling is classified into three types: in-plant recycling, post-industrial recycling (PIR), and post-consumer recycling (PCR). To effectively reduce plastic waste in the future, post-consumer recycling must be expanded. When polymer materials distributed on the market are recycled, the characteristics of the recycled material are generally significantly different from the virgin material. This is because materials change over time depending on the thermal history during the initial molding of the product and the environment in which they are used over the years, and different materials can become mixed when they are used as composite materials or due to insufficient separation processing during the recycling process. For the above reasons, when using polymeric materials, it is necessary to promptly evaluate their creep deformation characteristics, predict their behavior under practical conditions, and use this information appropriately in product design. It is therefore becoming increasingly important to predict long-term creep deformation properties using theoretical analyses of limited experimental data obtained in short study periods.

Creep deformation behavior can generally be classified into three stages. In the primary creep region of the initial deformation, deformation proceeds quickly at first, and after that, deformation is suppressed by strain hardening and the strain rate decreases. In the secondary creep stage, deformation proceeds at a nearly constant rate while maintaining a small deformation rate; then, in the tertiary stage, damage to the material becomes apparent, deformation accelerates, and this leads to fracture. Many studies have been conducted to model such creep deformation behavior, and a well-known creep model is the power law model [3,4,5]. It has long been known that the power law model can well represent the creep characteristics of various materials. However, a simple power law model has limitations, such as being unable to represent phenomena up to tertiary creep. Since then, many creep models have been proposed and used industrially, with improvements and development persisting (e.g., see review articles [6,7]). The modeling of polymer material creep properties has also been developed by applying the results of other materials such as metals and rocks. A notable characteristic of the creep deformation properties of polymer materials is that the temperature dependence and stress dependence of the deformation rate can be expressed through Eyring’s law [8], the details of which were reviewed by Starkova et al. [9]. In addition, since the deformation behavior of polymer materials includes both elastic responses associated with the entanglement and elongation of molecular chains and viscous responses associated with the mutual movement (translational movements) of molecular chains, it is necessary to treat them as viscoelastic material. The damping behavior over time associated with viscoelastic properties is generally expressed using an exponential function. The exponential function has the very useful property of having a consistent mutual connection with complex functions and trigonometric functions. For this reason, linear viscoelasticity theory has been developed based on the relaxation or retardation properties described by the exponential function, and the nonlinear viscoelasticity theory on which it is based [10]. However, due to the basic molecular structure and composition state, the relaxation and retardation characteristics of polymeric materials cannot be described using a single exponential function, and it is necessary to generalize the model by superposing multiple relaxation or retardation times. In our previous studies on polymer processing, we modeled the melt viscoelasticity of polymeric materials and used it in simulations, but it was necessary to consider multiple relaxation time distributions to represent transient flow response. It was not only for elastic materials with long relaxation times [11,12] but also for polypropylene (PP) in injection molding [13], which has a relatively short relaxation time and high fluidity. As a result, the number of parameters used increases, which is one of the drawbacks of viscoelastic flow analysis. Even in polymer solids, when the time scale of interest is very wide, such as creep, it becomes necessary to consider multiple retardation times.

The fractional derivative viscoelastic model is effective in addressing these issues. Since fractional derivatives can be defined mathematically, they have been applied to the analysis of viscoelasticity, starting with Gemant’s 1/2 derivative Maxwell model [14]. It was later discovered that the viscoelastic response can be described with a small number of parameters if the appropriate differential order is set using the fractional differential viscoelastic model. The analysis of Scott Blair [15] and subsequent research on fractional differential viscoelastic models (e.g., Smit [16]) have provided a basis for the previously experimentally obtained power law model of creep, while the validity of its application to various problems has also been discussed [17,18]. Related research has thus been ongoing for many years, and in the last decade or so, increasing attention has been drawn toward modeling the mechanical properties of various materials using fractional differential viscoelastic models.

The importance of creep evaluation and prediction of polyolefin materials has become significant with the industrialization of polyethylene pipes. Polyethylene pipes have been researched and industrialized since the 1950s, and their use has become widespread due to advances in materials development and the prediction of long-term creep failure based on the Larson-Miller method [19]. On the other side, polypropylene is widely used in structural parts for automobiles, home appliances, etc., and there is more importance in predicting creep deformation and failure according to the loading conditions and shape effects of each product. The modeling of the creep properties of solid polypropylene has been studied mainly by applying the power law model [20,21,22,23], while the application of temperature–time [24,25,26] and stress–time conversion [27] has also been discussed. Blends of reinforcement materials such as fiber reinforcement can improve creep properties if the adhesion at the interface with the filler is good, and many modeling studies have been reported [28,29,30,31,32,33]. It has been reported that adding fillers is particularly effective in improving the creep properties of recycled PPs [34]. On the other hand, it has been reported that blending soft components to impart impact resistance deteriorates creep properties [35,36,37]. A macroscopic general purpose constitutive law has been derived based on microscopic deformation mechanisms such as polymer network deformation and the damage phenomena, and good prediction results have been obtained for the creep behavior of polypropylene [38]. In recent years, the creep phenomenon of polypropylene has been simulated using a microscopic coarse-grained model [39]. These studies have great implications for understanding the creep behavior of polypropylene microscopically. On the other hand, for practical use in engineering, it is useful to describe the phenomenon with as few parameters as possible using a simple model, even if it is specialized only for a creep phenomenon. The Burger model is often used to predict creep because the meaning of the model parameters is clear, and there are many examples of PP analysis [37,38,40]. Kurt et al. analyzed the effect of molecular weight and molecular weight distribution on the short-term creep of polypropylene using the Burger model and obtained reasonable results [41]. However, the differences in long-term creep between experiments and calculations using the Burger model were also reported [42]. It was improved by setting multiple retardation times using the generalized Kelvin–Voigt model, but the empirical power law model still better represented the phenomenon [43]. With such research results, attempts have been made to apply a time-dependent power law model to PPs as well, based on the concept of fractional differential viscoelasticity [44,45,46].

In this study, the behavior of various polypropylenes, including recycled PPs, from primary creep to tertiary creep under various temperatures and stresses was evaluated. Based on the experimental results, a phenomenological model was developed based on fractional viscoelastic calculus to evaluate the creep properties of each material. The Monkman–Grant law is known to be useful in predicting creep rupture [9], and in this study, the prediction of the rupture time using the Monkman–Grant law was performed using the calculated minimum strain rate obtained by the prediction model.

## 2. Experimental

### 2.1. Materials

Three types of virgin PPs and three types of recycled PPs were used as model materials. One type of homo PP and two types of impact-resistant PPs copolymerized with ethylene were selected as virgin PPs. The homo PP used was hPP-1 (F-704NP, Prime Polymer Co., Ltd., Tokyo, Japan), which is for film application. The impact-resistant PPs used were low-rigidity hiPP-1 (J-466HP, Prime Polymer Co., Ltd., Tokyo, Japan) and high-rigidity hiPP-2 (J704UG, Prime Polymer Co., Ltd., Tokyo, Japan), both of which are for injection molding. The recycled PPs used were rePP-1 (sorted as a mixed type), rePP-2 (a soft type), and rePP-3 (a hard type). All of these were recycled from polypropylene products collected from the market. Although these recycled products undergo careful sorting and a meticulous process to remove foreign matter, it is inevitable that minute amounts of impurities may be mixed in. In addition, ethylene copolymerized PP was also assumed to be mixed, and it is thought that it contains a certain amount of ethylene or polyethylene components. As shown in Table 1, the MFR of these materials is on the same order of magnitude. Test pieces for creep tests were injection molded from these materials. An injection molding machine FNX80 (Nissei Plastic Industrial Co., Ltd., Nagano, Japan) was used to prepare dumbbell test pieces (JIS K7139 A1 [47]). The dumbbell test pieces are 3 mm thick, with a distance between the marked lines of 80 mm and a width between the marked lines of 10 mm. The injection molding was carried out under the following conditions: cylinder temperature 220 °C, mold temperature 40 °C, injection rate 50 cm^3^/s, and holding pressure 40 MPa. The tensile modulus evaluated using these test pieces at room temperature and a test speed of 50 mm/min is shown in Table 1.

### 2.2. Creep Test

**Figure 1:**
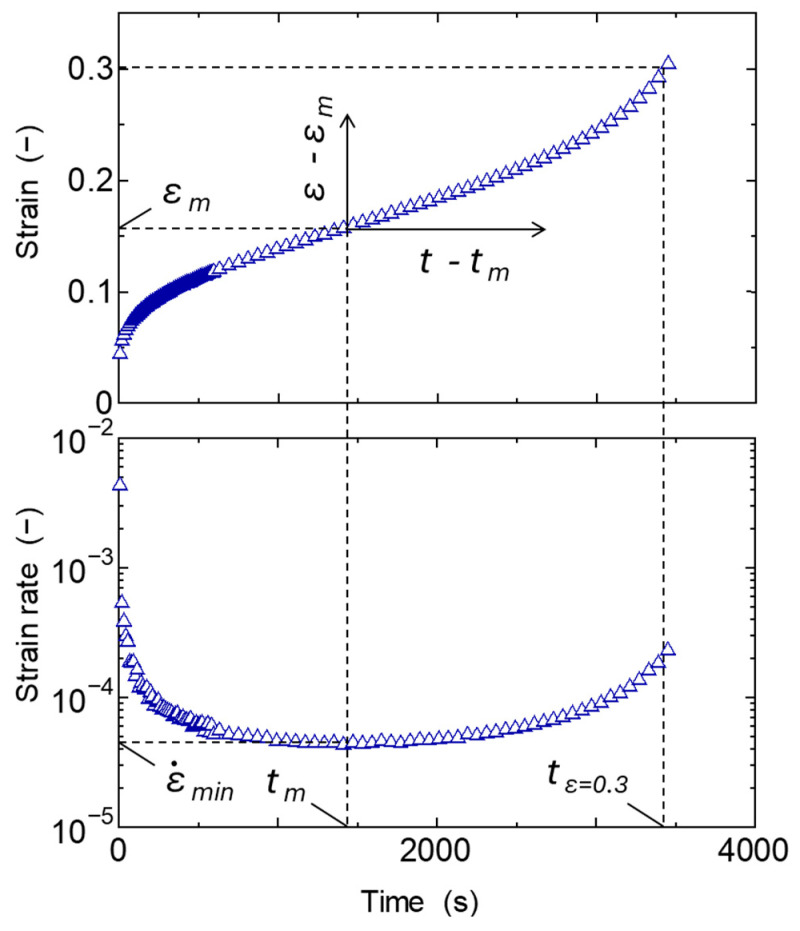
Experimental results of strain and strain rate transition of rePP-1 at 50 °C, 12 MPa. Evaluation items are indicated.

Tensile creep tests were conducted on the prepared test pieces. The creep tester used was C300-3 (Toyo Seiki Seisaku-sho, Ltd., Tokyo, Japan). The stress level was set between 2 MPa and 16 MPa depending on the material. The test time was set at 1000 min as standard, but tests up to 15,000 min were also conducted under some conditions. Three test temperatures were set: 40 °C, 50 °C, and 60 °C. It is possible to set higher test temperatures, but it has been reported that in the case of PP, when the temperature exceeds 60 °C, the aging process progresses faster than the creep deformation rate and it becomes inconsistent with low-temperature creep [48]. For this reason, one method is to perform creep tests after achieving thermal stability via annealing, but it has been reported that the temperature and time-equivalent shift factor still change around 60 °C [49]. To clarify the characteristics of general untreated injection-molded products, creep evaluation was conducted on injection-molded products without annealing. However, to avoid the effect of a large increase in crystallinity in the short term after molding [50], creep tests were performed at least 48 h after molding at room temperature, as is commonly done when evaluating other mechanical properties. Creep tests were conducted until the nominal strain reached 0.3. Since the amount of strain was relatively low, evaluations were consistently conducted at the nominal stress and strain in this study. Figure 1 shows the progress of creep strain and strain rate for rePP-1 at 50 °C and 12 MPa. The initial increase in strain due to primary creep, the secondary creep process in which the strain increases monotonically, and the tertiary creep process in which the increase in strain accelerates from around 3000 s can be confirmed. The evaluation items from the experimental results, which will be explained in the following section, are shown in the figure.

## 3. Results and Discussion

### 3.1. Analysis from Primary to Secondary Creep

#### 3.1.1. Experimental Results and Modeling Using Various Creep Models

**Figure 2:**
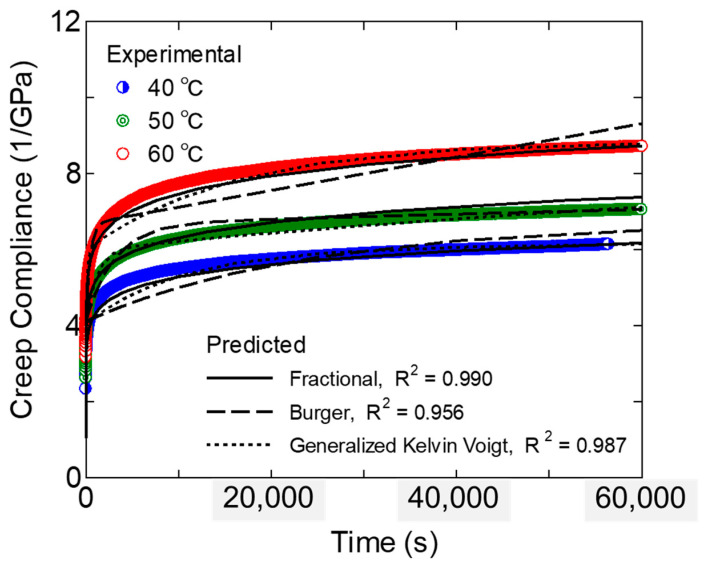
Experimental results of strain and strain rate transition of rePP-1 at 50 °C, 12 MPa. Evaluated values are indicated.

**Figure 3:**
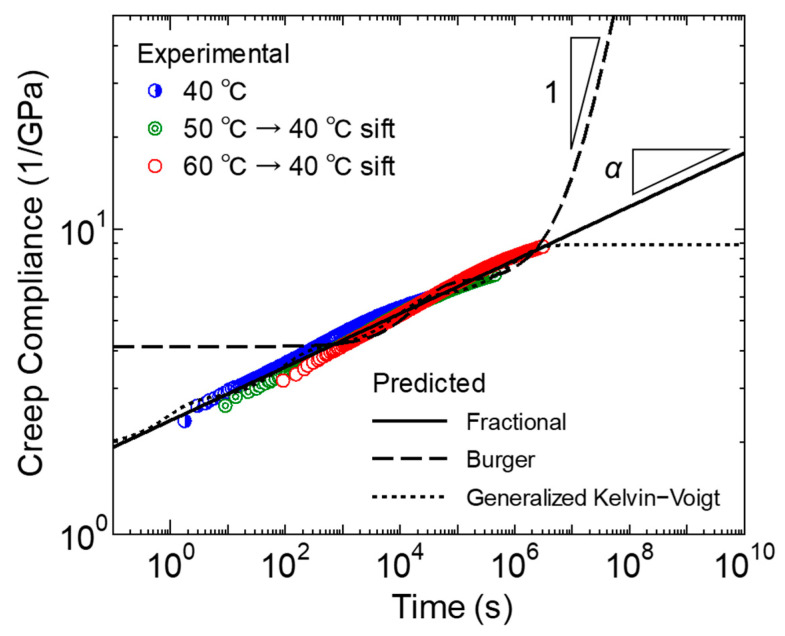
Experimental and predicted results of the 40 °C master curve of the transient creep compliance (rePP-1, 4 MPa).

As the first step in the analysis of the experimental results, the primary and secondary creep behaviors were analyzed at low-stress creep. Figure 2 shows the results of evaluating the time dependence of creep compliance at various temperatures at 4 MPa for rePP-1. It is confirmed that the deformation does not reach the tertiary creep stage where deformation accelerates toward rupture at any temperature, and the deformation rate continues to decrease with time. It can also be shown that the mobility increases with increasing temperature, and the creep strain increases. It has been widely reported that the temperature and stress dependence of the creep strain rate $\dot{\varepsilon }$ for polymer materials follows Eyring’s law [8].

(1) $$\dot{\varepsilon }={\dot{\varepsilon }}_{0}\;\exp\left(-\frac{E_{a}}{RT}\right)\sinh\left(\frac{\sigma V_{a}}{k_{B}T}\right)$$

where *T* is the absolute temperature, *σ* is the stress, *E_a_* is the activation energy, *V_a_* is the activation volume, *R* is the gas constant, *k_B_* is the Boltzmann constant, and ${\dot{\varepsilon }}_{0}$ is a coefficient. Since the stress is constant at 4 MPa, a time shift is performed based on the Arrhenius equation in the first half of Equation (1), and a master curve is created.

(2) $$a_{T}=\exp\left[-\frac{E_{a}}{R}\left(\frac{1}{T}-\frac{1}{T_{ref}}\right)\right]$$

where *a_T_* is the shift factor and *T_ref_* is the reference temperature. Figure 3 shows the results as a log–log plot. It was confirmed that the master curves for primary and secondary creep could be established in this study using the time-temperature conversion law, as has been reported in many reports.

**Figure 4:**
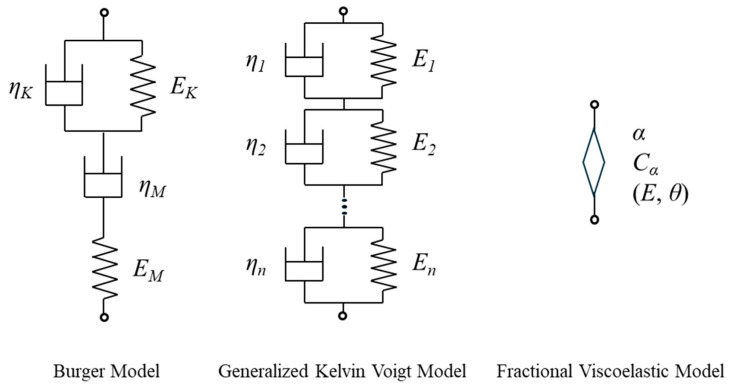
Models used in prediction of primary and secondary creep behavior.

We then attempted to express this master curve using a typical creep model, as shown in Figure 4. The time dependence of creep strain under constant stress conditions using the Burger model is expressed by the following formula.

(3) $$\varepsilon \left(t\right)=\frac{\sigma }{E_{M}}+\frac{\sigma t}{\eta_{M}}+\frac{\sigma }{E_{K}}\left[1-exp\left(\frac{-E_{K}\;t}{\eta_{K}}\right)\right]\;$$

*E_M_* and *η_M_*, respectively, denote the elastic modulus and viscosity of the Maxwell model part in the Burger model. *E_K_* and *η_K_*, respectively, denote the elastic modulus and viscosity of the Kelvin–Voigt model part.

(4) $$\varepsilon \left(t\right)=\sum_{i=1}^{n}\frac{\sigma }{E_{i}}\left[1-exp\left(\frac{-t}{\tau_{i}}\right)\right]$$

where *E_i_* and *τ_i_* are the elastic modulus and retardation time of the Kelvin–Voigt element of the *i*-th retardation mode, respectively. According to the fractional differential viscoelastic model, the response of stress to strain is defined as follows [16]:

(5) $$\sigma \left(t\right)=E\theta^{\alpha }\frac{d^{\alpha }\varepsilon \left(t\right)}{{dt}^{\alpha }}=C_{\alpha }\frac{d^{\alpha }\varepsilon \left(t\right)}{{dt}^{\alpha }},\;0\;≦\;\alpha \;≦\;1\;$$

where *E* is the elastic coefficient, *θ* is the retardation time, and *α* is the order of fractional derivative. When *α* is 0, this formula corresponds to a perfect elastic body, and when it is 1, it corresponds to a purely viscous body. When *α* is between zero and 1, it shows a viscoelastic response, and *α* can be an index of the magnitude of the viscous properties. Using the Riemann–Liouville fractional derivative, the *α* fractional derivative of Equation (5) can be expressed as follows:

(6) $$\frac{d^{\alpha }\varepsilon \left(t\right)}{{dt}^{\alpha }}=\frac{1}{\Gamma \left(1-\alpha \right)}\int_{0}^{t}{\left(t-\tau \right)}^{-\alpha }\varepsilon \left(\tau \right)d\tau$$

where Γ represents the gamma function.

(7) $$\Gamma \left(m\right)=\int_{0}^{\infty }e^{-\tau \alpha }\tau^{m-1}d\tau$$

By substituting Equation (6) into Equation (5) and integrating under constant stress, the following creep equation is obtained [16]:

(8) $$\varepsilon \left(t\right)=\frac{\sigma }{E\;\theta^{\alpha }}\frac{t^{\alpha }}{\Gamma \left(1+\alpha \right)}=\frac{\sigma }{C_{\alpha }}\frac{t^{\alpha }}{\Gamma \left(1+\alpha \right)}\;,\;0\;≦\;\alpha \;≦\;1$$

Figure 4 shows the representation of the components of the three models described above. Using these models, model parameters were obtained to optimize the fitting of the master curve in Figure 3. For optimization, the Adam gradient descent method was used, and iterative calculations were performed to minimize the square error between the experimental and calculated values of creep strain. Table 2, Table 3 and Table 4 show the parameters optimized for each model. Figure 3 shows the calculation results of the master curve using these parameters. Figure 2 shows the calculation results at each temperature obtained by temperature–time conversion from the master curve, and the numerical values of the coefficient of determination. As can be seen from Figure 3, the master curve of the creep diagram maintains a linear relationship over a wide range of time scales. It was difficult to express this phenomenon using the Burger model, which has only one retardation time, and in the model, the linear relationship with a constant gradient can only be expressed in a specific region, and large errors occurred in the startup of creep deformation. It has been reported that the short-term creep strain transition of polypropylene can be well expressed through the Burger model [41], but this study also confirmed that it is difficult to describe long-term primary and secondary creep [43]. On the other hand, in the generalized Kelvin–Voigt model, the linear relationship can be expressed over a wide range of time scales by setting multiple retardation times. However, the number of parameters increases. In this study, the retardation time is set approximately every two decades, but 10 model parameters are still required. In contrast, according to the fractional differential viscoelasticity model, the linear relationship of the creep diagram can be very well expressed using only two model parameters, *C_a_* and *α*. The power law model originally represents the experimental linear relationship of the log–log creep function, and the fractional derivative coincides with this. Furthermore, when focusing on a longer time scale than employed in the experimental results, the Burger model, in which the viscous response becomes dominant toward the end, eventually has a gradient that approaches 1. In the generalized Kelvin–Voigt model, in which the longest retardation time responds last, and the viscous properties disappear, the final gradient approaches zero. On the other hand, the fractional derivative viscoelastic model is advantageous for extrapolation, as it shows a constant creep diagram gradient even on the longer time scale. The same results can be obtained by the conventional power law creep model. However, a simple power law model does not provide information on the viscoelastic response. Considering the scope of future research, the power law function description by the fractional differential viscoelastic model is more effective for polymeric materials that show viscoelastic response.

Based on the above results, in this study, a fractional derivative viscoelastic model was used to represent the transition from primary creep to secondary creep. To more accurately represent the primary to secondary creep of polymer materials, the application of fractional derivative models to more complex ones such as the Maxwell [51], Kelvin–Voigt [52], Zener [53], and Burger [54] models has been considered. In this study, we also considered extending the fractional derivative viscoelastic model to the Maxwell model to more accurately represent the initial elastic response. This is equivalent to the Findley model [5], a creep model that provides good phenomenological prediction results. However, this extension only slightly improves the error of the calculated results compared to our experimental results, so we prioritized simplicity and used a single fractional derivative viscoelastic model here.

#### 3.1.2. Temperature and Stress Dependence of Fractional Differential Order

**Figure 5:**
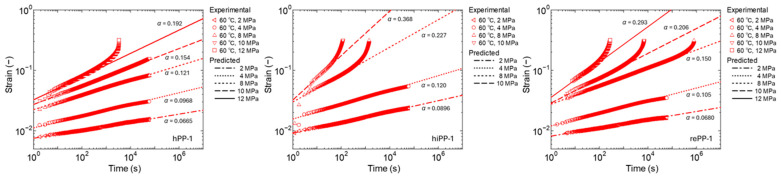
Experimental results of stress dependence of creep strain curves at 60 °C and prediction results up to secondary creep using the fractional viscoelastic model.

**Figure 6:**
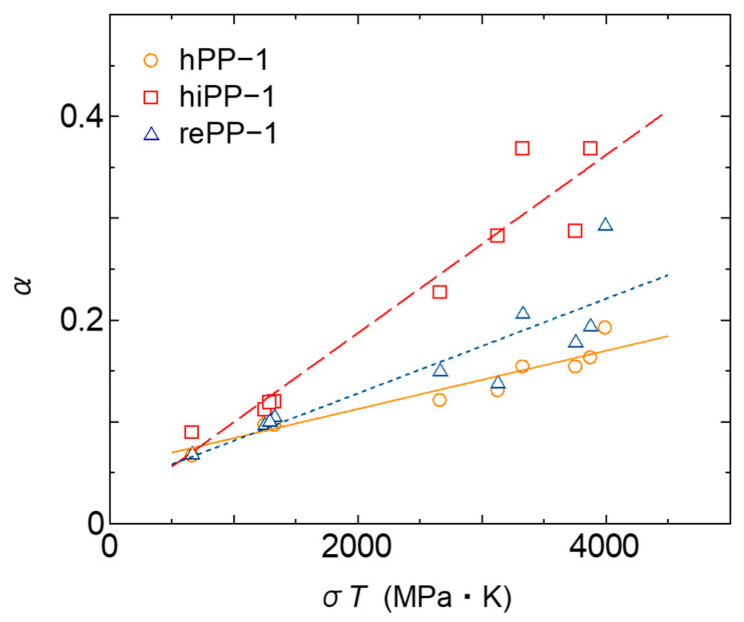
Relationship between the product of stress and temperature *σT* and fractional order α.

The behavior of primary and secondary creep when stress is changed was investigated in this study. Figure 5 shows the experimental results of creep diagrams when stress is changed at 60 °C for the three PPs. For creep in low-stress fields, the creep diagrams show a linear log–log relationship within the range of experimental results. As the stress increases, creep deformation accelerates and the slope increases from a certain point, i.e., confirming a transition to tertiary creep. The stress at which the transition to tertiary creep occurs depends on the stiffness of each material. For the high-stiffness hPP-1, the linear relationship is maintained even at 10 MPa, while for the low-stiffness hiPP-1, the tertiary creep can be confirmed at 8 MPa. rePP-1 shows an intermediate response, so no specificity of the recycled material was confirmed in the primary and secondary creep behavior, and the response was in accordance with the stiffness of the material. Note that the 8 MPa for rePP1 is a special experimental result measured up to about 10^6^ s. The creep diagrams maintain a linear relationship until the tertiary creep is reached. We therefore considered the linear region on the short time scale to be the primary and secondary creep region and fitted it with the fractional differential viscoelastic model of Equation (8). The resulting fractional differential order, i.e., the slope *α* of the line, is shown in Figure 5. As indicated by Wang et al. [46], it can be confirmed that the slope increases as the stress increases for any material. In this study, it was confirmed that the slope *α* of the temperature dependence also increases as the temperature increases. The above results show that it is difficult to obtain a straight master curve of the first- and second-order creep behavior via a horizontal shift with respect to time based on the Eyring law or a vertical shift with respect to the amount of strain. In this study, we therefore attempted to express the stress dependence and temperature dependence of *α* based on the experimental results. Initially, a function based on the Eyring type function of Equation (1) was set, but it could not express it well. After various considerations, we therefore set the following linear function as an experimental formula and made it as simple as possible, with the product of stress and temperature as explanatory variables:

(9) $$\alpha \;\left(T,\sigma \right)=A\;\sigma T+B$$

where *A* and *B* are material constants. The results of predicting *α* using this formula for three types of materials are shown in Figure 6. It is confirmed that the change in *α* with stress and temperature can be expressed relatively well. This empirical formula was created with a focus on simplification based on current experimental results, but there may be room for further development based on supplementary experimental results and future theoretical investigations.

### 3.2. Analysis of Tertiary Creep

#### 3.2.1. Application of the Monkman–Grant Law

**Figure 7:**
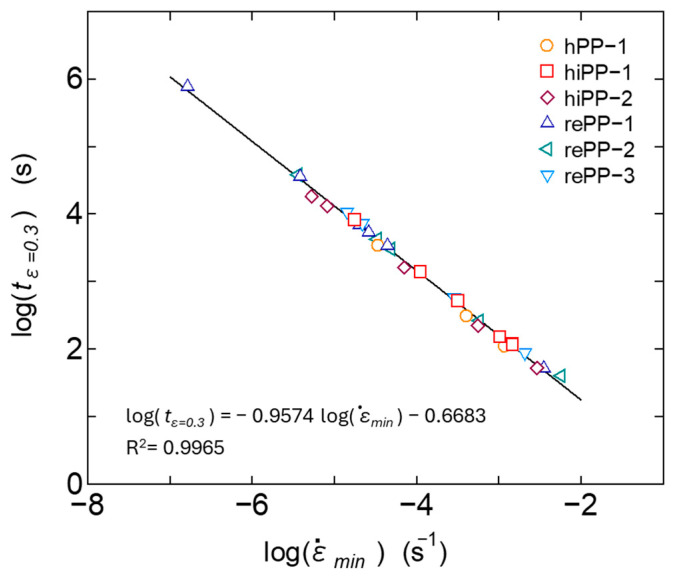
Evaluation results based on Monkman–Grant law when the evaluation target is the time at the 0.3 strain.

**Figure 8:**
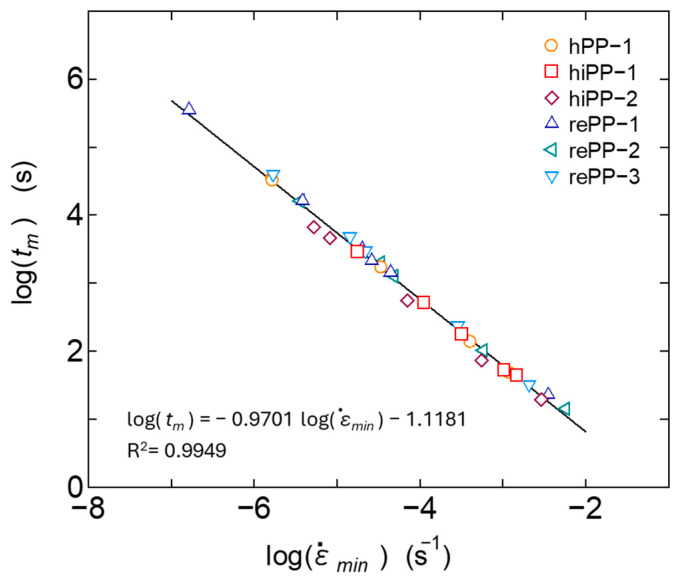
Experimental results of the relationship between the minimum strain rate and the time to reach the minimum strain rate.

The secondary creep process is interpreted as a process in which deformation progresses at a nearly constant, gentle strain rate, due to the equilibrium between the suppression of deformation due to strain hardening of the viscoelastic response of the primary creep process and the acceleration of plastic deformation due to the progression of damage in the tertiary creep process. However, the strain rate changes slowly in the secondary creep process and shows a minimum value as shown in Figure 1. It is known that there is a certain correlation between this minimum value ${\dot{\varepsilon }}_{min}$ and the rupture time, and when expressed as a log–log curve, the two are in a linear relationship; i.e., the Monkman–Grant law holds. It has been reported that this relationship also holds for polymer materials and their composite materials [9,55]. The relationship between the minimum strain rate ${\dot{\varepsilon }}_{min}$ and the time *t_r_* at which the strain reaches 0.3 was therefore investigated in relation to our experimental results in which the creep strain reached its maximum value among the creep test results of each material measured at various temperatures and stresses. The results are shown in Figure 7. It was confirmed that all materials, including homo PP, impact-resistant PPs, and recycled PPs, follow the same linear relationship of the Monkman–Grant law. When the same Monkman–Grant law is shown, it can be interpreted that the deformation mechanism from secondary creep to tertiary creep is similar. From the above results, it is suggested that in addition to homo PP, impact-resistant PPs copolymerized with ethylene, and recycled PPs that have been recovered from the market and are thought to be adversely affected by mixtures and foreign matter, the deformation in the tertiary creep process in the range of strain up to 0.3 proceeds by almost the same mechanism. Amjadi [55] reported that regrind HDPE, a recycled product used in factories, also exhibits the same Monkman–Grant law as virgin material. In this study, the post-consumer recycled PPs also show similar results to virgin ones up to the relatively small strain range. Figure 8 shows the relationship between the minimum strain rate ${\dot{\varepsilon }}_{min}$ and the time *t_m_* at which this minimum rate occurs. It can be confirmed that this relationship also has a good correlation with the Monkman–Grant law. The above results suggest that if ${\dot{\varepsilon }}_{min}$ is known, the creep deformation behavior from the secondary to tertiary creep can be accurately predicted.

#### 3.2.2. Stress Dependence on Tertiary Creep

**Figure 9:**
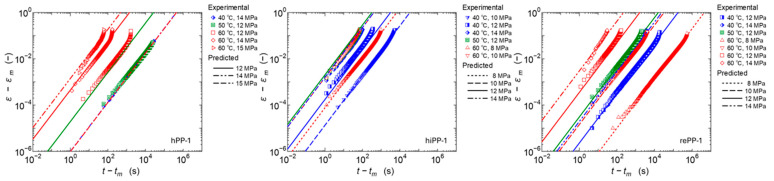
Experimental and predicted results of the relationship between time and strain growth from the point of minimum strain rate.

**Figure 10:**
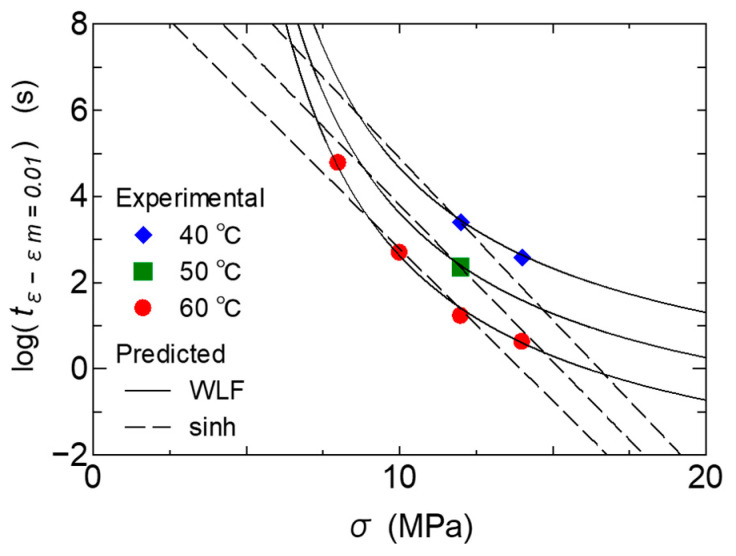
Experimental results of the relationship between stress and time when strain increases by 0.01 from the point of minimum strain rate, and the predicted results based on Eyring’s law.

**Figure 11:**
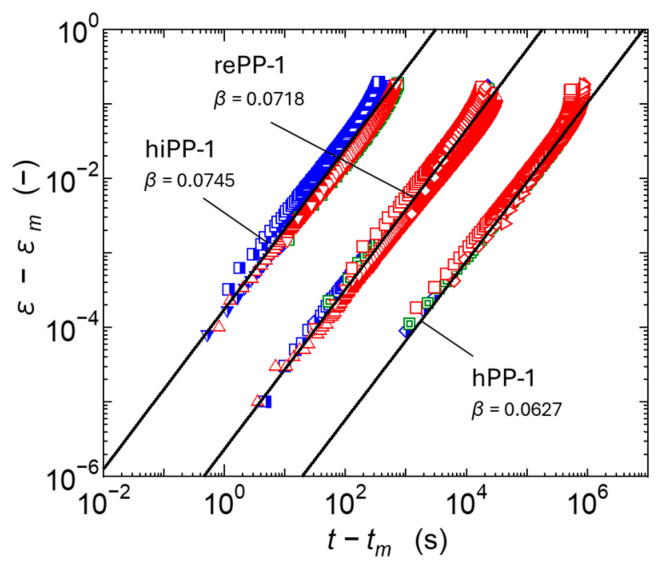
Experimental and predicted results of the master curve of the relationship between time and strain growth from the point of minimum strain rate. The experimental results are shown with the same symbols as in Figure 9, and the solid lines represent the calculated results.

The point of minimum strain rate is the moment when plastic deformation due to damage becomes dominant, and it can be said that from this point on, the development of tertiary creep begins to surface. In this study, we therefore investigated the creep deformation behavior when the time point of the minimum strain rate is used as the base point. Figure 9 shows the relationship between time and strain for three types of PPs, starting from the time *t_m_*. For each material, it can be confirmed that the increase in strain in the log–log plot changes linearly with the same slope. In other words, the strain increases exponentially at a rate of progression that is different from that of the primary and secondary creep. This result suggests that the master curve can be obtained for the growth of creep in the tertiary creep process by shifting the time with temperature and stress. Starting from the time of the minimum strain rate, the time until the strain reaches 0.01 was therefore evaluated, and the relationship with stress was examined with temperature as a parameter. The results are shown in Figure 10. Furthermore, the shift factor was calculated based on Equation (1) expressed as a sinh function for stress and the Eyring law with the WLF type function for the stress [9]; the results of calculating the time f or 0.01 strain are shown in the same figure.

(10) $$\dot{\varepsilon }={\dot{\varepsilon }}_{0}\;exp\left(-\frac{E_{a}}{RT}\right)\exp\left[\frac{C_{1}\left(\sigma -\sigma_{0}\right)}{C_{2}+\sigma -\sigma_{0}}\right]$$

where *C*_1_, *C*_2_, and *σ*_0_ are material constants. As can be seen from the figure, The sinh function that gives a linear relationship could not express the change in creep growth rate with the change in stress. On the other hand, the WLF type function could express the calculation results, which show a downward convex curve with respect to stress, well. Next, we attempted to create a master curve by shifting the experimental results in Figure 9 using this WLF-type function. The results are shown in Figure 11. By setting a WLF-type function for stress for all three materials, master curves could be created. It has been reported that the stress shift in many polymer materials follows a WLF-type function [9]. In this study, it was validated that the stress dependence of the tertiary creep process of PP also follows the WLF formula. Here, the WLF type model was selected placing emphasis on consistently good prediction accuracy for various materials, as shown above. However, as shown in Figure 10, when graphing one material, the number of data is still insufficient, and further verification of the validity of the model is recommended. The WLF-type Eyring Equation (10) expresses how the change in free volume due to a change in stress contributes to mobility. One possible reason why the relationship between the two is not linear is thought to be because of stress-induced changes in the crystal structure of PP. However, this nonlinear relationship has also been confirmed in amorphous resins [56]. This may need to be discussed from the perspective of the strain rate dependence of strain hardening in polymer viscoelasticity. A future challenge is to confirm the manifestation of the stress dependence of the creep phenomenon and the changes in the microscopic structure and to clarify the mechanism by which the stress dependence is manifested.

From the above results, it is suggested that the tertiary creep process can be expressed using a power law model shifting the time with respect to stress and temperature, as in the case of the primary and secondary creep processes. The extension of the power law model to express the tertiary creep is often used, and it has also been expressed using a fractional derivative viscoelastic model. However, the increase in deformation of tertiary creep accompanied by damage is large, and the index value is larger than 1 [57,58]. This indicates that the mobility exceeds that of a pure viscous body with an index value of 1 and goes beyond the concept of a fractional derivative viscoelastic model. In this study, we therefore treat it as a power law pure viscosity model [59]. Considering the physical meaning of the parameters, we set up a model in which the viscosity *η*(t) decreases over time due to damage depending on the stress and temperature as follows:

(11) $$\eta \left(t\right)={a_{T,\sigma }\eta }_{0}{\left(\frac{t}{a_{T,\sigma }}\right)}^{-\beta }$$

where *a_T,σ_* is a shift factor with temperature and stress based on Equation (10), and *η*_0_ and *β* are material constants. The larger *β* is, the more the viscosity decreases over time, and *β* can be interpreted as an index of how easily damage progresses. This model can be considered as an extension of the description in which the damage itself is expressed as a power law with respect to time [60]. Calculating stress using Equation (11) gives Equation (12), and integrating this formula under constant stress gives Equation (13).

(12) $$\sigma =\eta \left(t\right)\dot{\varepsilon }\left(t\right)=a_{T,\sigma }\eta_{0}{\left(\frac{t}{a_{T,\sigma }}\right)}^{-\beta }\dot{\varepsilon }\left(t\right)$$

(13) $$\varepsilon \left(t\right)=\frac{\sigma }{{\left(1+\beta \right)\eta }_{0}}{\left(\frac{t}{a_{T,\sigma }}\right)}^{1+\beta }$$

The tertiary creep line calculated using the above formula is shown in Figure 11. Furthermore, the calculation results at each temperature and stress are shown in Figure 9. The value of *β* is also shown in Figure 11, and it is confirmed that *β* is a positive value and the viscosity decreases with time.

### 3.3. Prediction of the Overall Creep Behavior

#### 3.3.1. Overall Creep Prediction Model

From analyzing the experimental results of primary, secondary, and tertiary creep, a creep deformation model is constructed to predict all processes. In general products, a large deformation in the tertiary creep stage is not permitted, and behavior up to secondary creep is important. From this point of view, it is considered important in practice to predict the minimum strain rate, and the amount of strain and time elapsed until the minimum strain rate is reached. In this study, a model was therefore set up that places emphasis on predicting these things. Equation (14) shows the model equation that represents the change in strain over time from initial creep to tertiary creep.

(14) $$\varepsilon \left(t\right)=\frac{\sigma }{E\;\Gamma \left[1+\alpha \left(T,\;\sigma \right)\right]}{\left(\frac{t}{a_{T,\sigma 1}\theta }\right)}^{\alpha \left(T,\;\sigma \right)}+\frac{\sigma }{\eta_{0}\left(1+\beta \right)}{\left(\frac{t}{a_{T,\sigma 2}}\right)}^{1+\beta }$$

(15) $$a_{T,\sigma 1}=\frac{exp\left(-E_{a}/RT_{ref}\right)\sinh\left(\sigma_{ref}V_{a}/k_{B}T_{ref}\right)}{exp\left(-E_{a}/RT\right)\sinh\left(\sigma V_{a}/k_{B}T\right)}$$

(16) $$a_{T,\sigma 2}=\frac{exp\left(-E_{a}/RT_{ref}\right)exp\left[C_{1}\left(\sigma_{ref}-\sigma_{0}\right)/\left(C_{2}+\sigma_{ref}-\sigma_{0}\right)\right]}{exp\left(-E_{a}/RT\right)exp\left[C_{1}\left(\sigma -\sigma_{0}\right)/\left(C_{2}+\sigma -\sigma_{0}\right)\right]}$$

The first term in Equation (14) represents the growth of viscoelastic strain that is dominant in the first and second creep processes, and the second term represents the growth of viscous strain accompanied by damage that is dominant in the tertiary creep process. The fractional differential index *α*(*T*, *σ*) depending on temperature and stress, is explained in Equation (9). Since *E* and *θ* are not uniquely determined when the *α* is constant, *C_a_* in Equation (5) was evaluated in Section 3.1. However, *E* and *θ* can be specified here because the variation in α is considered, and these were evaluated as material parameters. Tertiary creep is often modeled using the power law, but modeling is often performed by setting a threshold value for stress and dividing the cases [59,61]. Alternatively, when using fractional differential models, it has become mainstream in recent years to use a variable order fractionation method to divide the cases and model them [62,63,64,65]. In this study, damage to polymer materials according to stress and temperature is assumed to occur immediately after loading, even if at an extremely slow rate, and is expressed as a simple superposition of viscoelastic strain and viscous strain, as in Equation (14). This expression makes the evaluation of the minimum strain rate possible. This is the strain rate at which the positive acceleration of strain due to damage accumulation in the tertiary creep process becomes dominant over the negative acceleration of strain in the primary and secondary creep processes. Differentiating Equation (14) with respect to time gives the following equation for calculating the strain rate.

(17) $$\dot{\varepsilon }\left(t\right)=\frac{\alpha \left(T,\;\sigma \right)\;\sigma \;}{E\;\Gamma \left[1+\alpha \left(T,\;\sigma \right)\right]{\left(a_{T,\sigma 1}\theta \right)}^{\alpha \left(T,\;\sigma \right)}}\;{t\;}^{\alpha \left(T,\;\sigma \right)-1}+\frac{\sigma }{\eta_{0}{{\;a}_{T,\sigma 2}}^{1+\beta }}\;{t\;}^{\beta }$$

Further differentiating this equation with respect to time gives the following equation for the acceleration of strain

(18) $$\ddot{\varepsilon }\left(t\right)=\frac{\alpha \left(T,\;\sigma \right)\;\left[\alpha \left(T,\;\sigma \right)-1\right]\;\sigma \;}{E\;\Gamma \left[1+\alpha \left(T,\;\sigma \right)\right]{\left(a_{T,\sigma 1}\theta \right)}^{\alpha \left(T,\;\sigma \right)}}{t\;}^{\alpha \left(T,\;\sigma \right)-2}+\frac{\beta \;\sigma }{\eta_{0}{{\;a}_{T,\sigma 2}}^{1+\beta }}\;{t\;}^{\beta -1}$$

When the strain rate is at a minimum, Equation (18) becomes zero, so *t_m_* can be analytically derived as follows:

(19) $$t_{m}={\left[\frac{\alpha \left(T,\;\sigma \right)\left[1-\alpha \left(T,\;\sigma \right)\right]\;{a_{T,\sigma 2}}^{1+\beta }\eta_{0}}{\beta E\;\Gamma \left[1+\alpha \left(T,\;\sigma \right)\right]{\left(a_{T,\sigma 1}\theta \right)}^{\alpha \left(T,\;\sigma \right)}}\right]}^{1/\left(1-\alpha \left(T,\;\sigma \right)+\beta \right)}\;$$

By substituting this value into Equation (17), the minimum strain rate ${\dot{\varepsilon }}_{min}$, can also be analytically determined as follows

(20) $${\dot{\varepsilon }}_{min}=\frac{\alpha \left(T,\;\sigma \right)\sigma }{E\;\Gamma \left[1+\alpha \left(T,\;\sigma \right)\right]{\left(a_{T,\sigma 1}\theta \right)}^{\alpha \left(T,\;\sigma \right)}}{t_{m}}^{\alpha \left(T,\;\sigma \right)-1}+\frac{\sigma }{\eta_{0}{a_{T,\sigma 2}}^{1+\beta }}{t_{m}}^{\beta }$$

The above model is for uniaxial extension, but by using von Mises equivalent stress, including that of Eyring’s law, for the stress in Equations (14)–(16), and uniaxial equivalent strain based on invariants for the strain tensor, it will be possible to extend the model to predict creep in general products with multiaxial stresses [8].

#### 3.3.2. Parameter Identification and Material Property Analysis

**Figure 12:**
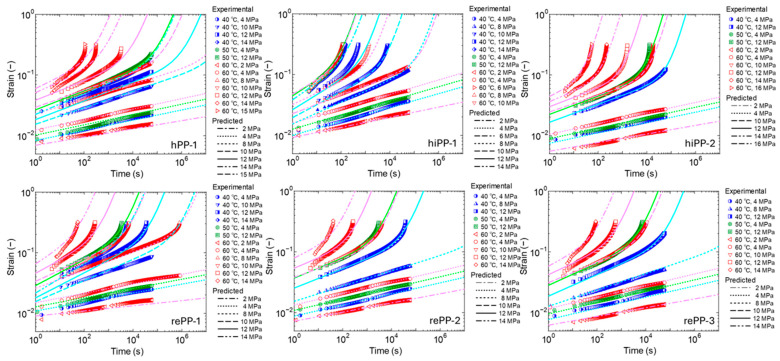
Comparison of experimental overall creep strain curves with predicted results using the presented creep model. The cyan, light green, and pink lines show the calculation results for 40 °C, 50 °C, and 60 °C, respectively.

Using the above model, the experimental results of the creep behavior of the model materials were predicted. The parameters were optimized using the least squares method, as in Section 3.1, and optimization was performed using the gradient descent method. However, since the target strain amount includes a wide range of order values, the mean square error of the logarithmically transformed strain value was evaluated so that the accuracy at small strain would not decrease. A comparison of the calculation results using optimized parameters and the experimental results is shown in Figure 12. The experimental results are plotted, and the calculation results are displayed as lines. Differences in stress in the calculation results are shown by changing the type of line. It can be confirmed that this model is able to adequately represent creep behavior in low-stress fields where primary and secondary creep dominates, as well as creep behavior in high-stress fields where tertiary creep occurs, and the temperature dependence of these behaviors, for all materials. However, at the end of the tertiary creep, the difference between the experimental results and the calculation results is confirmed. The experimental results show steep strain growth, whereas the experimental results do not follow this, and this tendency is especially true in hPP-1. However, it was observed that the specimens were beginning to the neck at the time, which indicates that uniform deformation has already been lost at this point and the application of creep models that assume uniform deformation is no longer valid. For this reason, data with strains exceeding 0.25 were excluded from the parameter fitting for all materials. The most significant necking was observed in hPP-1, which is thought to be due to hPP-1 having a strong crystal structure, a small yield strain, and a high yield stress, which results in a large stress difference between the upper and lower yield points.

Table 5 shows the results of the optimized model parameters and the coefficient of determination for optimization. The reference temperature *T_ref_* and the reference stress *σ_ref_* are set at 40° C and 8 MPa, respectively. If these values change, only the time-related parameters *θ* and *η*_0_ will change. The coefficient of determination shows that all materials were fitted with high accuracy. For the stress shift due to Eyring’s law, when the WLF-type equation was applied to the primary and secondary creep as in the case of the tertiary creep, the stress dependency could not be expressed. For the primary and secondary creep, stress dependency could be expressed well by using the sinh function, which enabled the evaluation of the activation volume. It has been reported that the activation volume of polypropylene at 60 °C is 3 to 8 nm^3^ [66], and the evaluation results obtained in this study were of the same order. However, only hiPP-2 had a clearly smaller activation volume value than the others. The reason is unclear, but it may be natural that the values vary due to the influence of various components added in the design of impact-resistant PPs, orientation accompanying molding processing, residual stress, etc. [67].

While stress dependence cannot be treated uniformly with creep from primary to tertiary, the phenomenon can be expressed well by setting the same activation energy in all processes for temperature. Initially, we attempted to optimize the activation energy by dividing it into multiple different values, but the results showed that the values of primary and secondary creep and tertiary creep were similar. It has been reported that the activation energies evaluated based on the mechanical properties of PP range from 210 to 370 kJ/mol [24], but it has also been reported that semi-crystalline PP has multiple relaxation processes, the phenomenon is not simple, and these mechanisms are expressed by multiple influences of the activation energies [49]. In this study, the activation energy could be treated relatively simply, and all these values were similar, around 200 kJ/mol, which may be because the temperature range set here is narrow. Complexity may be apparent if setting a higher or lower temperature. However, it is practically useful if the temperature dependence is simple at 60 °C or lower, which is an important temperature range used for many PP products. Looking at the differences between the model materials, the activation energy of the homo PP hPP-1 was large, and the values of hiPP-1 and hiPP-2, which contain ethylene, were small, at around 180 kJ/mol. Three types of recycled PPs showed intermediate values between these.

Next, looking at the elastic modulus *E* and retardation time *θ* of the fractional differential viscoelastic model from primary to secondary creep, the results for *E* were all of the same order, about 0.1 to 0.2 GPa. On the other hand, *θ* varied greatly depending on the material. This is thought to be because the movement of molecules behaving viscoelastically in the amorphous part has a large effect on *θ*, which represents the retardation in the viscoelastic response. The PP used here is of the same order as MFR, so there is thought to be no significant difference in molecular weight, but there is a large difference in crystallization characteristics, and there is thought to be a large difference in the thickness of the amorphous phase, the number of tie molecules, and the tension state. It is thought that these factors cause a large difference in the retardation time *θ*. Focusing now on the parameters of the tertiary creep, *η*_0_, which indicates viscous properties, a similar trend to the above-mentioned *θ* is exhibited, suggesting that it is influenced by the composition and structure of the material. Of interest is the parameter *β*, which represents the progression of the damage; the parameter corresponds to activation energy, with hPP-1 being the smallest, and hiPP-1 and hiPP-2 having the largest values of around 0.5. Three types of recycled PPs showed intermediate values between these. Homo PP is thought to be less likely to cause damage because its crystallinity is high and crystal structure is strong. On the other hand, impact-resistant PP is designed to be prone to irreversible deformation such as localized structural damage and molecular slippage by copolymerizing ethylene to enhance its energy absorption effect, and it appears that these factors have a detrimental effect on creep deformation. The recycled PP was again found to be somewhere between homo PP and impact-resistant PP. From the above, it can be said that recycled PP contains a certain quantity of components with high crystallinity and a strong crystal structure derived from homo PP and shows a creep resistance similar to homo PP. Note that the evaluation result of *β* here is different from the value quantified in Section 3.2.2. This is because the onset of tertiary creep is not evaluated based on the time of the minimum strain rate here, and *β* is optimized to best represent the entire creep curve.

In conclusion, regarding creep behavior with relatively low deformation, recycled PP exhibits intermediate properties between homo PP and impact-resistant PP, and the specificity of recycled PPs has not been particularly confirmed. Although the quality of recycled PPs is diverse and cannot be generally known, the above results suggest that even recycled PPs recovered from the market can deliver the same performance as virgin material regarding static creep properties if the product is carefully designed and handled.

#### 3.3.3. Application to Predict Rupture

**Figure 13:**
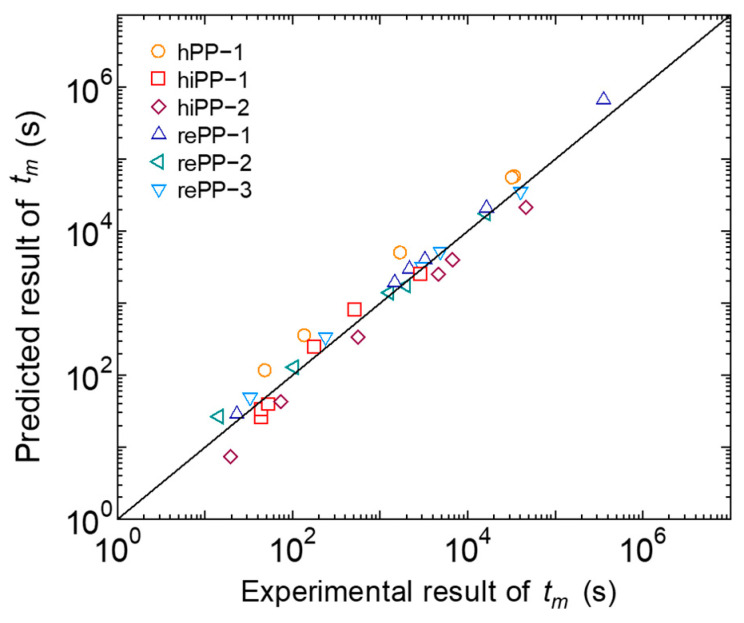
Relationship between experimental and predicted time at minimum strain rate.

**Figure 14:**
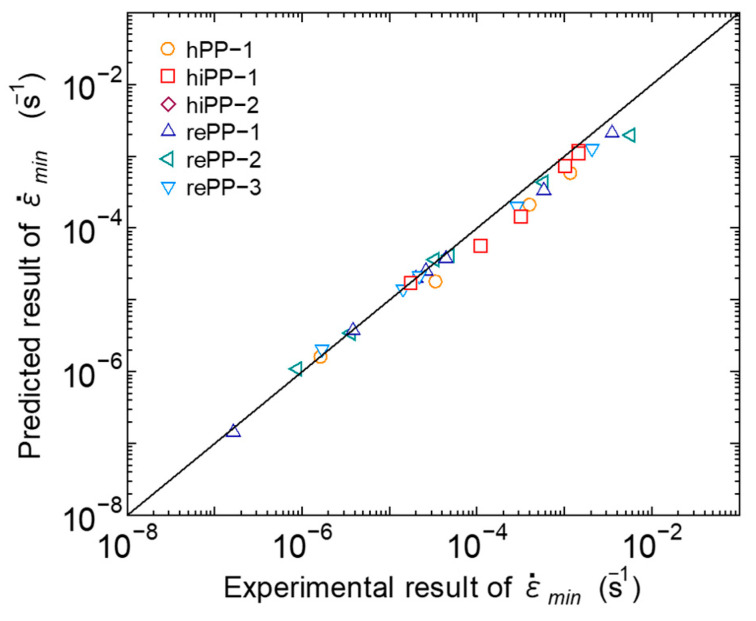
Relationship between experimental and predicted minimum strain rate.

For the experimental results in which the minimum strain rate was observed, the relationship between the calculation results and the experimental results of the time *t_m_* to reach the minimum strain rate and the minimum strain rate ${\dot{\varepsilon }}_{min}$ was investigated. Figure 13 shows the relationship between the predicted results of *t_m_* calculated using Equation (19) and the experimental results. Figure 14 shows the relationship between the calculation results of ${\dot{\varepsilon }}_{min}$ calculated using Equation (20) and the experimental results. For *t_m_* in Figure 13, a certain amount of error occurred between the experimental results and the calculation results. On the other hand, for ${\dot{\varepsilon }}_{min}$ in Figure 14, the experimental results could be predicted well via calculation. These results are due to the extremely small fluctuation in the strain rate in the second creep stage, as shown in Figure 1. The results in Figure 13 show that there is room for improvement by further refining the prediction model. However, for ${\dot{\varepsilon }}_{min}$, which is a key factor, the temperature dependence and stress dependence can be predicted well for each material, so it is possible to predict the fracture time using the Monkman–Grant law in Figure 7 based on the calculation results. For *t_m_*, the correlation with ${\dot{\varepsilon }}_{min}$ has also been experimentally confirmed using the Monkman–Grant law, as shown in Figure 8. It has been confirmed that these relationships are almost on the same line regardless of the PP grade, so the prediction model in this study can also be used to predict *t_m_* and ${\dot{\varepsilon }}_{min}$ for new PP materials. Furthermore, the ability to predict the transition in the amount of strain until ${\dot{\varepsilon }}_{min}$ is reached using Equation (14) is believed to be useful in designing PP products that take creep resistance characteristics into account.

## 4. Conclusions

Based on fractional differential viscoelastic calculus, a model was developed to predict the primary to tertiary creep in the tensile creep deformation of various polypropylenes. While the Burger model has limitations in its ability to express a wide range of time scales and the generalized Kelvin-Voigt model requires many parameters, the fractional differential viscoelasticity model can predict the creep strain growth behavior of PP from the primary to secondary creep with few parameters. It was confirmed that the fractional differential order in the primary and secondary creep processes increases with increasing stress and temperature dependence, and this can be expressed using an empirical formula. In the tertiary creep, the strain grows according to a power law at a faster growth rate different from the primary and secondary creep, which can be expressed well by treating it as a pure viscous body with damage. The target temperature range was 40 to 60 °C, and in this region, the temperature dependence of creep deformation can be handled relatively simply, and the same creep strain of around 200 kJ/mol could be set for all materials in all creep deformation processes. On the other hand, for stress dependence, the Eyring law of the sinh function could be applied to the primary and secondary creep processes, and the activation volume could be specified, but for the tertiary creep, it was necessary to adopt a WLF-type shift function. A strain growth model was established by simply superimposing the primary and secondary creep strains from the above-mentioned fractional differential viscoelastic model and the tertiary creep strain from a pure viscous model involving damage, and this enabled good prediction of the experimental results for the total strain growth behavior of the six types of PP studied here. Regarding the differences between the model materials, the creep growth rate during the primary and secondary creep processes was shown to correspond to the rigidity of each material. In the tertiary creep, it was confirmed that homo-PP, with its strong crystal structure, showed slight damage progression, whereas impact-resistant PPs with energy absorption properties due to microscopic fracture showed rapid damage progression. The three types of recycled PPs collected from the market showed intermediate properties between these virgin PPs, and the recycled PPs used in this study showed no peculiarities as recycled products. It was confirmed that the creep experimental results for all materials, including the recycled PPs, fell on the same straight line of the Monkman–Grant law. The creep model developed in this study is advantageous in that it cannot only predict the change in creep strain but also easily analytically determine the minimum strain rate, which is an important index for predicting the entire creep phenomenon. The application of the Monkman–Grant law is effective in predicting the long-term creep characteristics of new PPs and applying them to various products. Future research directions include the verification of this model under a wide range of conditions and accumulating data on materials, including the types of recycled PPs, molding methods, and conditions for test pieces, and the evaluation of conditions such as long-term evaluation and temperature conditions. Furthermore, the challenge is to improve the present model as necessary based on the accumulated data.

## Figures and Tables

**Table 1:** Model materials.

Name	MFR/g/10 min (230 °C, 2.16 kg)	Tensile Modulus/MPa (50 mm/min)	Type
hPP-1	7.0	1150	Homo PP
hiPP-1	3.1	800	Ethylene copolymerized PP
hiPP-2	5.0	1250	Ethylene copolymerized PP
rePP-1	5.7	950	Recycled PP (PCR)
rePP-2	6.8	990	Recycled PP (PCR)
rePP-3	4.0	1070	Recycled PP (PCR)

**Table 2:** Model parameters (Burger model).

*E_M_*/GPa	*η_M_*/GPa·s	*E_K_*/GPa	*η_K_*/GPa·s
0.243	1,220,000	0.391	9290

**Table 3:** Model parameters (generalized Kelvin–Voigt model).

*τ_i_*/s	*E_i_*/GPa
0.01	1.942
1	0.808
100	1.294
10,000	1.941
1,000,000	2.878

**Table 4:** Model parameters (fractional viscoelastic model).

*C_α_*/GPa·s *^α^*	*α*
0.443	0.0876

**Table 5:** Optimized model parameters of the presented creep model for the polypropylenes.

Parameters		hPP-1	hiPP-1	hiPP-2	rePP-1	rePP-2	rePP-3
Activation Energy	*E_a_*/kJ/mol	218.8	188.0	180.5	201.4	212.8	203.0
Primary–Secondary Creep Parameters							
Activation Volume	*V_a_*/nm^3^	2.977	3.328	1.349	2.402	3.646	2.268
Elastic Modulus	*E*/GPa	0.1211	0.09815	0.2129	0.1592	0.1039	0.1861
Retardation Time	*θ*/s	1.480 × 10^6^	1.654 × 10^4^	2.224 × 10^4^	1.452 × 10^4^	6.431 × 10^5^	1.365 × 10^4^
Parameter determining fractional order *α*	*A*	2.776 × 10^−5^	5.265 × 10^−5^	2.495 × 10^−5^	5.314 × 10^−5^	2.720 × 10^−5^	3.409 × 10^−5^
	*B*	0.04898	0.04804	0.05419	0.007178	0.05706	0.04933
Tertiary Creep Parameters							
Parameters of WLF type stress shift function	*C*_1_	60.94	39.79	52.41	57.90	45.25	70.32
	*C*_2_/MPa	4.232	3.829	8.122	1.516	1.172	1.748
	*σ*_0_/MPa	2.369	2.456	0.005922	4.374	5.362	2.792
Viscosity	*η*_0_/GPa·s	7.977 × 10^9^	1.538 × 10^6^	1.062 × 10^10^	8.866 × 10^8^	1.403 × 10^8^	2.63 × 10^8^
Damage Index	*β*	0.1702	0.4357	0.5317	0.2729	0.1958	0.242
Coefficient of Determination R^2^		0.995	0.991	0.997	0.995	0.999	0.990

## Data Availability

The original contributions presented in this study are included in the article. Further inquiries can be directed to the corresponding authors.
